# The effect of a structured medication review on quality of life in Parkinson's disease: The study protocol

**DOI:** 10.1016/j.conctc.2018.100308

**Published:** 2018-11-28

**Authors:** N.G.M. Oonk, K.L.L. Movig, E.M. Munster, K. Koehorst-Ter Huurne, J. van der Palen, L.D.A. Dorresteijn

**Affiliations:** a Department of Neurology, Medisch Spectrum Twente, Enschede, the Netherlands; b Department of Clinical Pharmacy, Medisch Spectrum Twente, Enschede, the Netherlands; c Department of Pulmonary Medicine, Medisch Spectrum Twente, Enschede, the Netherlands; d Pharmacy De Hofbraak, Haaksbergen, the Netherlands; e Department of Epidemiology, Medisch Spectrum Twente, Enschede, the Netherlands; f Department of Research Methodology, Measurement, and Data Analysis, University of Twente, Enschede, the Netherlands

**Keywords:** Parkinson's disease, Therapy adherence, Quality of life, Medication review, Medication, SMR, structured medication review, DRP, drug related problem, CDS, chronic disease score, MID, minimally important difference, PDQ, Parkinson's disease questionnaire, ALDS, Amsterdam Medical Center linear disability score, EQ-5D-5L, EuroQOL-5 Dimensions-5 Levels, NMS-Quest, non-motor symptoms questionnaire, VAS, Visual analogue scale

## Abstract

**Background:**

Treatment of Parkinson's disease (PD) is symptomatic and frequently consists of complicated medication regimes. This negatively influences therapy adherence, resulting in lower benefit of treatment, drug related problems and decreased quality of life (QoL). A potential effective intervention strategy is a structured medication review, executed by community pharmacists. However, little is known about the effects on clinical endpoints like QoL, as well as on feasibility and cost-effectiveness in PD patients.

**Objectives:**

To assess the effect of a structured medication review on QoL in PD patients. Secondary objectives are measurements of physical disability, activities in daily life, non-motor symptoms, health state, personal carers' QoL and cost-effectiveness. Furthermore, a better insight in the process of performing medication reviews will be obtained from the perspective of community pharmacists.

**Methods:**

In this multicenter randomized controlled trial we aim to enroll 200 PD patients from the outpatient clinic of three Dutch hospitals. Community pharmacists will perform a structured medication review in half of the assigned patients; the other half will receive usual care. Data obtained by use of six validated questionnaires will be collected at baseline and after 3 and 6 months of follow-up. Semi-structured interviews with community pharmacists will be conducted till data saturation has been reached.

**Discussion:**

This trial targets a high-risk patient group for whom optimizing therapy by a structured medication review might be of added value. If effectiveness is proven, this could further promote the implementation of pharmaceutical care in a primary care setting.

## 1 Introduction

Parkinson's disease (PD) is a progressive, neurodegenerative and age-related disease, affecting approximately 7.5 million patients worldwide. Because of the aging population, this is expected to increase up to more than 9 million in 2030 [1,2].

Most of the PD symptoms result from degeneration of dopaminergic neurons within the substantia nigra, leading to a shortage of dopamine in the striatum [3]. Clinical characteristics are tremor, rigidity, bradykinesia and postural instability [4,5]. Other non-motor symptoms, both dopaminergic and non-dopaminergic, include progressive cognitive and autonomic dysfunction. PD symptoms contribute to the reduction of functional abilities, shortened life expectancy and reduced quality of life (QoL) [[6], [7], [8]]. The clinical signs occur across all PD stages, but become increasingly prevalent as the disease progresses.

To date, drug treatment is the mainstay for its management [9,10]. However, the effectiveness of long-term treatment is known to decrease, leading to end-of-dose deterioration, fluctuations in medication response and side-effects [11,12]. When the disease progresses and the therapeutic window narrows, dosing frequency will increase and other medicines need to be added. Simultaneously, the total medication amount frequently rises due to comorbidities [13,14]. Patients often face complicated medication schedules and keeping track of it can be a challenging task [10,[15], [16], [17], [18]].

Medication adherence is an important factor towards success of treatment [19], although consistent adherence in PD is on average merely 50% [16,[20], [21], [22], [23], [24], [25], [26]]. This notably contributes to a reduced QoL and an increased burden on the healthcare system [14,15,19,20,27,28].

One of the barriers influencing compliance include complicated medication regimens with multiple daily dosing [20]. Polypharmacy easily leads to missing doses, and drug related problems (DRPs) occur [[29], [30], [31], [32], [33]].

To both improve adherence and optimize therapy, an often-recommended method and potentially effective intervention strategy is a structured medication review (SMR), defined as ‘a structured, critical examination of a patient's medicines with the objective of reaching an agreement with the patient about treatment, optimizing the impact of medicines, minimizing the number of DRPs and reducing waste’ [34].

The effects of an SMR, conducted by pharmacists, have been studied in all settings of care, where it has shown to reduce potentially inappropriate drugs [[35], [36], [37]] and to improve medication adherence, among others since customized care and shared decision-making may increase as patient preferences can be taken into account [38,39]. Furthermore, the amount of DRPs decreases [40,41]. In PD, performing an SMR may reduce the dosing frequency and complexity of medication schedules, possibly leading to simplified dosing and improved adherence [34,42], although not much is known about the effect of SMRs on clinical endpoints like QoL, which is an important parameter from a patient's perspective.

This manuscript describes a randomized controlled study assessing whether an SMR leads to improved QoL in PD patients. Additionally, measurements of daily life activities and physical disability, non-motor symptoms and personal carers' QoL will be performed. Furthermore, the feasibility and cost-effectiveness in this setting will be analyzed and a better insight in the process of performing medication reviews will be obtained from the perspective of community pharmacists.

## 2 Methods

### 2.1 Design

**Fig. 1:**
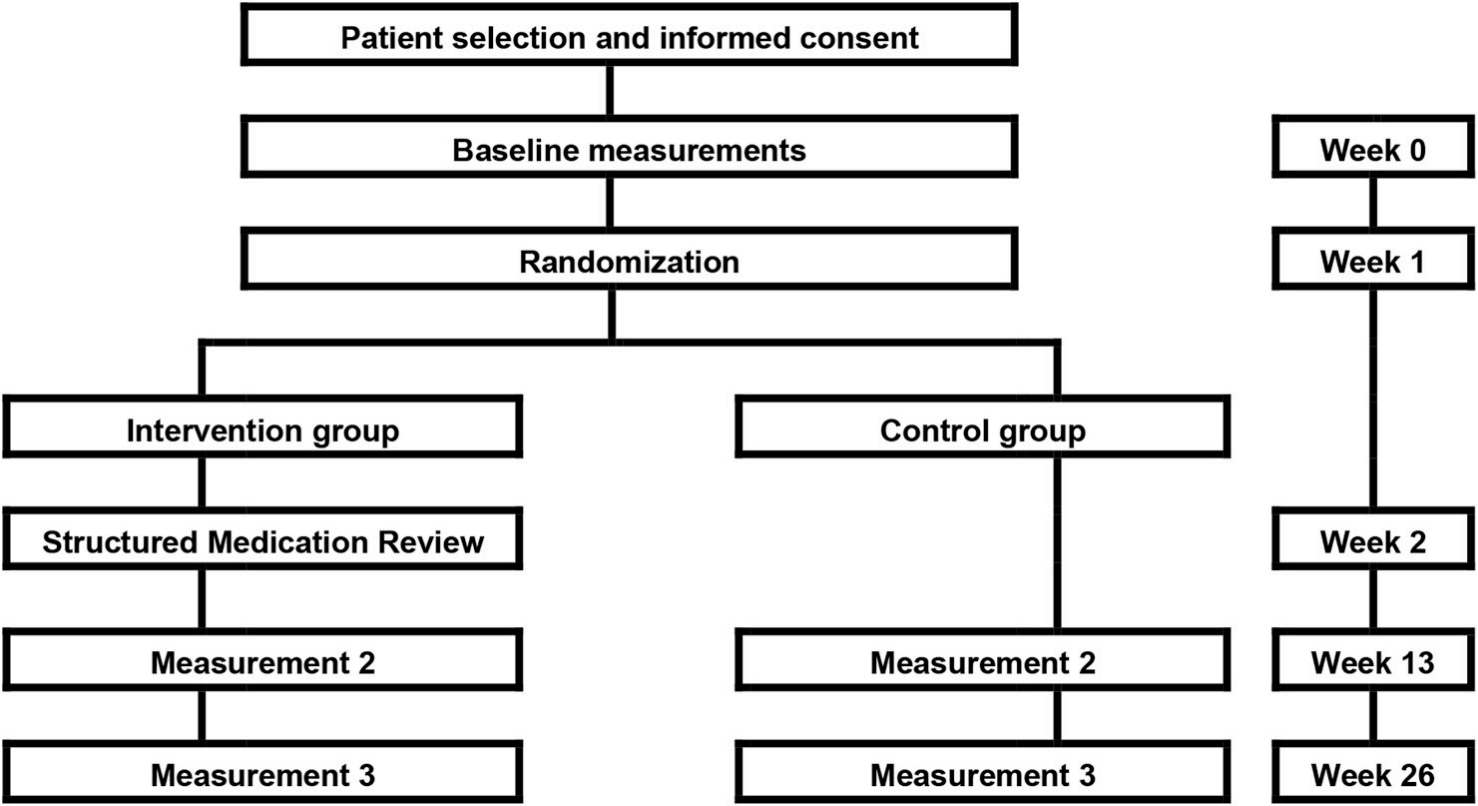
Study design flowchart.

The study is designed as a multicenter randomized controlled trial (RCT) of a medication review intervention as one-time assessment in PD patients. We aim to enroll 200 patients with idiopathic PD from the neurology department of 3 Dutch hospitals (Medisch Spectrum Twente, Ziekenhuis Groep Twente and Isala hospital). Data obtained by use of validated questionnaires will be collected at baseline and after 3 and 6 months of follow-up (Fig. 1).

The study will be conducted in agreement with the principles of the Helsinki Declaration and in accordance with the Medical Research involving Human Subjects Act (WMO). The research protocol is registered in the Dutch clinical trial register (NTR4500) and has been approved by the Medical Ethical Review Board Twente, the Netherlands.

### 2.2 Recruitment, consent and randomization

In order to be eligible to participate, patients must meet the following criteria: (1) diagnosed with PD according to the UK-brain banking criteria [43]; (2) ≥ eighteen years of age; (3) ≥ four different medications daily; (4) ≥ four medication intake moments daily; (5) expressing motor and non-motor symptoms; (6) living (semi)-independent in the region of Enschede, Almelo, Hengelo or Zwolle; (7) be able to read and write the Dutch language. Exclusion criteria are: (1) being unable to administer their own medication, e.g. when requiring assistance from medical home care. PD patients receiving help from personal or family caregivers were not excluded based on this criterium; (2) having received a medication review within a year prior to the study; (3) having received a Deep Brain Stimulator, continuous duodopa gastro-intestinal gel therapy or continuous apomorphine therapy within a year before the study or willing to receive this within three months.

As soon as informed consent is obtained by the investigator and baseline measurements are performed, patients will be randomly assigned in a 1:1 ratio with a blinded blocked randomization with block sizes of four and eight. Inclusion is definite when patients in the intervention group complete the baseline measurements and receive an SMR, while patients in the control group only need to complete baseline measurements.

### 2.3 Intervention

Community pharmacists will perform the SMR at the start of the study within the intervention group, while the control group will receive usual care and will not have a medication review during the next six months. All pharmacists are offered an accredited training regarding PD and drug treatment, the study protocol and the execution of an SMR beforehand. The latter is based on the Dutch Systematic Tool to Reduce Inappropriate Prescribing (STRIP)-method [44], a standardized tool for pharmacists for executing SMRs in Dutch patients. This method highly emphasizes the importance of involving the patient as a full partner in evaluating all medicines on drug-drug interactions, adverse reactions and problems with dosing or drug use. Possible barriers to adherence and non-adherent behavior are identified by asking questions in a non-judgmental way [45,46]. Possible interventions will be suggested, taking the patient's preferences, views and beliefs about their medicines in full account.

To ensure continuity of care and to facilitate collaboration between health care providers, clear documentation is required, for which a protocol is chosen based on the action plan for medication assessment of the Multidisciplinary guideline Polypharmacy in the Elderly and the abovementioned STRIP-method [44] (see Appendix). The protocol foresees the possibility of consulting a General Practitioner, neurologist and other specialists if necessary. Any adjustments in the medication regime will be communicated and explained to the patient.

After the review has been performed, all pharmacists record their proposed medication modifications or interventions, recommendations and possible other findings. This is sent to the collaborating health care providers. Together with the patient, the applied interventions will be evaluated within 4 months either by telephone or face-to-face.

### 2.4 Outcome measures

Next to demographic characteristics, information on medication will be obtained from patients and verified by their pharmacists. Furthermore, the Rx-Risk Comorbidity Score will be calculated and used as a comorbidity measure [47]. PD severity is measured by Hoehn & Yahr (HY) stage at inclusion, based on information regarding actual HY stage as noted in the electronic patient file [48]. In case of uncertainty, a verification was asked from the treating neurologist.

#### 2.4.1 Primary outcome

The primary outcome will be disease-specific quality of life, measured by the Parkinson's Disease Questionnaire-39 (PDQ-39). This validated and reliable instrument has 39 items comprising eight domains of health status: mobility, activities in daily living, emotional wellbeing, stigma, social support, cognitions, communication and physical discomfort [49,50]. It can be used to assess effectiveness of treatment, in which dimension scores are coded on a scale of 0–100 with a higher score indicating lower QoL [8]. A difference of 1.6 points in the PDQ-39 total score is required to pinpoint a clinically relevant difference or minimally important difference (MID) [51]. Depending on domain, MIDs vary between 1.8 and 11.4 points.

#### 2.4.2 Secondary outcomes

Secondary objectives include activities in daily life and physical disability (AMC Linear Disability Scale (ALDS) [52]; non-motor symptoms (Non-Motor Symptoms Questionnaire (NMS-Quest) [53,54] and health status (EuroQOL-5 Dimensions-5 Levels (EQ-5D-5L, including Visual Analogue Scale (VAS) [55,56]. By using the index-based values of the EQ-5D-5L, quality adjusted life years (QALY) can be calculated, necessary for assessing the cost-effectiveness of an intervention [55]. Costs need to be taken into account to calculate these QALYs and to compare the cost-effectiveness of the SMR with other interventions in patients with PD. Costs incurred during this study are costs of medication, hospitalization, outpatient visits and home care, and the costs associated with the SMR. To assess the effects on QoL for personal or home caregivers, the PDQ-Carer questionnaire will be used [57].

All SMR forms will be collected to analyze modifications in medication regime and recommendations given to patients. This will be used descriptively.

Semi-structured, face-to-face interviews with community pharmacists will be conducted after at least one SMR has been performed by the pharmacist, to obtain insight in probable bottlenecks in the process of performing medication reviews. Interviewing will take place till data saturation has been reached.

### 2.5 Sample size

Using the PDQ-39, a difference of 1.6 points in the overall score of this questionnaire is considered as clinically relevant [51]. In the current study, a difference of four points in the total score is expected to appear after intervention. A standard deviation of 15 was found in previous research [51]. This standard deviation is used to make a preliminary calculation of the sample size. Based on a significance of 5% and a power of 80%, a sample size of 198 subjects, separated into two groups of 99, is required to detect a clinically important difference between both groups. However, as the standard deviation might be different and due to the fact that the actual effect of a medication review is yet still unknown, an interim analysis will be performed after the inclusion of 2*25 patients and 3 months of follow up. This estimate will provide a more appropriate basis to determine the final sample size.

### 2.6 Safety reporting

All adverse events, defined as any undesirable experience occurring to a subject during the study, whether or not considered related to the experimental intervention, reported spontaneously by the subject or observed by the investigator or staff, will be recorded. Serious adverse events (SAEs) which are likely to be associated with the study intervention will be reported within 7 days after the responsible investigator has first knowledge of it. Due to the disease status and the advanced age of patients with PD included in this study, expected SAEs such as hospitalization and death may occur. Unlikely is that these SAEs are associated with participation in the study. To cover this, a six-monthly line listing will be provided to the Medical Ethical Review Board Twente. An annual safety report will be submitted to the accredited METC as well. A Data and Safety Monitoring Board was not installed.

### 2.7 Data analysis

Between-group differences in categorical variables will be analyzed using a Chi-square test or Fisher's exact test, as appropriate, while continuous variables will be analyzed using t-tests or a Mann-Whitney test, as appropriate. Continuous data will be presented as means with standard deviations (SD) in case of normal distributions or as medians with interquartile ranges (IQR) in non-normal distributions. For all continuous variables, a repeated measurement analysis will be performed to deal with possible missing data. According to the PDQ-39 manual, missing data will be imputed using Expectation Maximization [58].

The approach of a modified intention-to-treat analysis will be used, which essentially means that to include patients in this study, patients in the intervention group need to receive an SMR after baseline questionnaires are completed, and patients in the control group need to complete baseline questionnaires after randomization.

Secondly, a sensitivity analysis will be performed to measure the effects of an SMR in patients in whom at least one medication regimen change was implemented or to whom at least one recommendation has been given.

Data will be analyzed using SPSS version 24. P-values ≤ 0.05 are regarded as statistically significant. Data concerning the semi-structured interviews will be imported in and analyzed with Atlas Ti version 8.

## 3 Discussion

The complexity of PD easily leads to compliance issues and DRPs. This calls for a structured strategy to monitor pharmacological care. Additionally, as no ‘one size fits all’ principle can be applied to treat PD, a patient-centered approach is required. In this trial, the impact of an SMR on QoL in PD patients will be investigated.

Although previous research in different settings of care assumes a positive effect of an SMR on health outcomes such as QoL and cost-effectiveness, contradictory results are found. Willeboordse et al. (2017) found no effect of a medication review concerning QoL in 518 geriatric patients and the chronic use of ≥1 prescribed drug [59]. One explanation might be the non-complexity of the selected patient group. More specific high risk patient groups should be targeted, for whom SMRs might be of added value [21,35,36,60]. A recent study by Marvin et al. (2018) showed that in an acute in-hospital setting, a combination of age, problematic polypharmacy and the presence of DRPs were the most important criteria to identify these high risk patients [37].

An SMR appears to be time consuming and labor intensive [14,35,61]. In the Netherlands, community pharmacists should conduct SMRs as part of standard care with only a low contractual remuneration by the covering health insurance companies [60]. The prevention of DRPs and safety of medicines have become an important discipline in health policy as well as in medical and pharmaceutical care. PD patients seem to be a logical target, given their complex medication regimen. This suggests that an SMR in these patients might be of high value. Nevertheless, not much is known yet about the effect of SMRs on clinical endpoints like QoL in this patient group. A pilot from Stuijt et al. (2017) found that multifaceted pharmacist-led interventions improved medication adherence in 23 PD patients, with a significant positive effect on QoL. The effect of separate interventions could not be assessed, although the best improvement of motor symptoms resulted from the addition of the SMR, performed by one co-located pharmacist in an outpatient PD clinic [62].

A strength in our study is that since patients are included together with their own community pharmacists, our results may be more generalizable. The training that community pharmacists are offered in order to standardize pharmaceutical care, might furthermore positively affect the quality of an SMR, as informed pharmacists might understand more about PD, its symptoms, complications and treatment, and how to respond to a patient's demand.

Considering the experience of previous studies in a similar setting, we choose to include PD patients with not only polypharmacy, but at the same time the need to take medicines at least 4 times daily, which is an extra factor contributing to complex medication regimens [24]. This may further select the high-risk patient group in need for optimizing therapy by an SMR.

In addition, since a cost-effectiveness analysis will be part of our study, the effects of an SMR in PD patients might eventually provide more evidence for further translation into practice.

Nevertheless, potential limitations according to the study protocol should be discussed. First, although justified by the nature of the intervention, randomization is not blinded. This might lead to increased awareness of medication use in the intervention group patients beforehand, whereby the effect of the intervention may be influenced. Also, due to the selection of patients, compliance in clinical trials often is substantially higher compared to actual practice [29].

Finally, with regard to a patient's HY-stage, the most accurate assessment would be physical evaluation. However, since patients are recruited by phone, this physical test would place an additional burden on the patient and their possible caregiver and might result in lower recruitment rates. Therefore, the HY-stage will be distracted from the electronic patient file in this research and in case of uncertainty verified by the treating neurologist.

Our trial has the support of the Royal Dutch Society for Pharmacy, which may help to promote the implementation of pharmaceutical care in this setting if the current study proves the effectiveness of an SMR in patients with PD.

## Ethics approval and consent to participate

Ethical approval for this study was provided by the Medical Ethical Review Board Twente, the Netherlands (reference number NL48661.044.14). Written informed consent will be obtained from all participants.

## Trial registration

The research protocol is registered in the Dutch clinical trial register: NTR4500.

## Funding

This work was supported by the Royal Dutch Society for Pharmacy.

## Consent for publication

Not applicable.

## Competing interests

The authors declare that they have no competing interests.

## Authors' contributions

NO: study concept and design, critical revision of manuscript for intellectual content. KM, EM, KKtH: study concept and design. JvdP, LD: study concept and design, critical revision of manuscript for intellectual content, supervision.

## Figures and Tables

**Figure:**
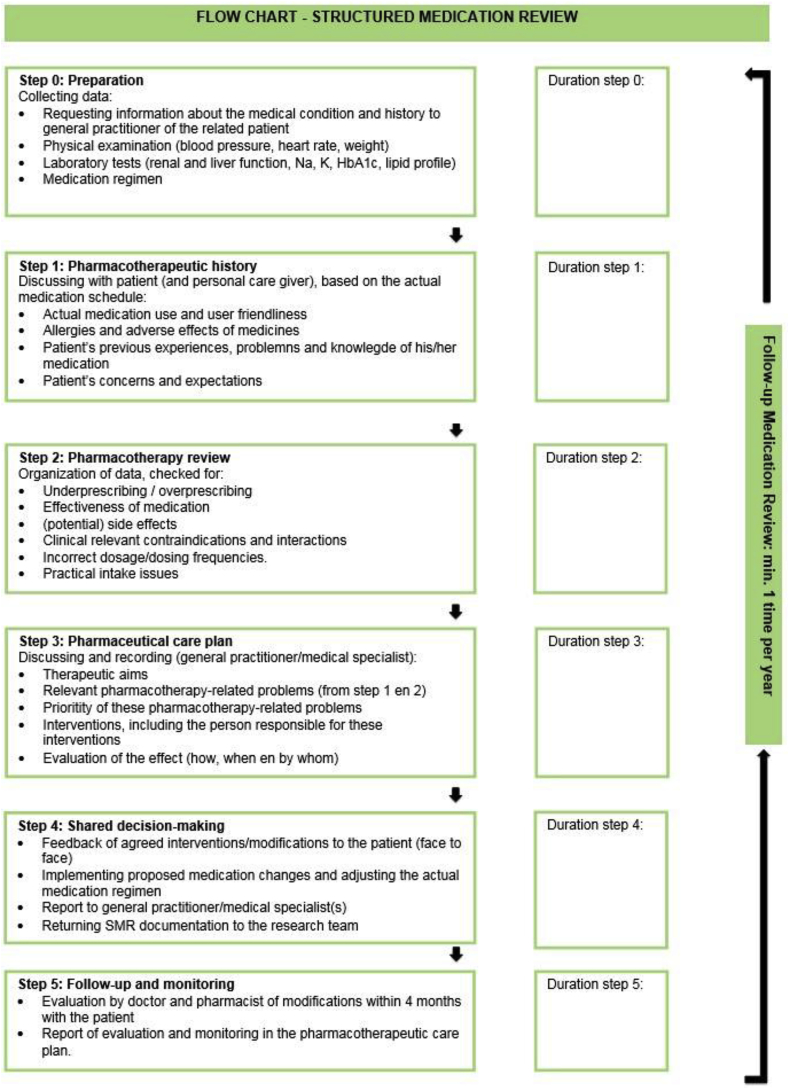


**Figure:**
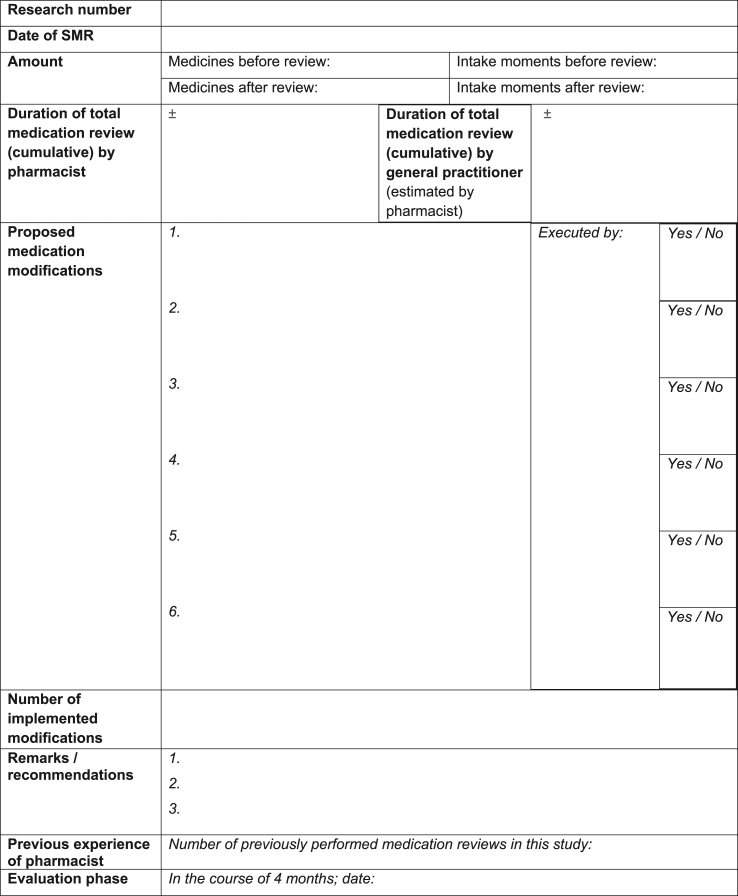


## Appendix A Supplementary data

Data Profile Data Profile
